# Robustness evaluations of pathway activity inference methods on gene expression data

**DOI:** 10.1186/s12859-024-05632-w

**Published:** 2024-01-12

**Authors:** Tay Xin Hui, Shahreen Kasim, Izzatdin Abdul Aziz, Mohd Farhan Md Fudzee, Nazleeni Samiha Haron, Tole Sutikno, Rohayanti Hassan, Hairulnizam Mahdin, Seah Choon Sen

**Affiliations:** 1 Soft Computing and Data Mining Center, Faculty of Computer Sciences and Information Technology, Universiti Tun Hussein Onn Malaysia (UTHM), 83000 Batu Pahat, Malaysia; 2 Computer and Information Sciences Department (CISD), Universiti Teknologi PETRONAS (UTP), 32610 Seri Iskandar, Malaysia; 3 Department of Electrical Engineering, Universitas Ahmad Dahlan (UAD), 55166 Yogyakarta, Indonesia; 4 Faculty of Electrical Engineering, Universiti Teknologi Malaysia (UTM), 81310 Johor Bahru, Malaysia; 5 Faculty of Computing, Universiti Teknologi Malaysia (UTM), 81310 Johor Bahru, Malaysia

**Keywords:** Pathway analysis, Reproducibility power, Robustness, PubMed text data mining, Literature validation, Pathway activity inference, Cancer classification

## Abstract

**Background:**

With the exponential growth of high-throughput technologies, multiple pathway analysis methods have been proposed to estimate pathway activities from gene expression profiles. These pathway activity inference methods can be divided into two main categories: non-Topology-Based (non-TB) and Pathway Topology-Based (PTB) methods. Although some review and survey articles discussed the topic from different aspects, there is a lack of systematic assessment and comparisons on the robustness of these approaches.

**Results:**

Thus, this study presents comprehensive robustness evaluations of seven widely used pathway activity inference methods using six cancer datasets based on two assessments. The first assessment seeks to investigate the robustness of pathway activity in pathway activity inference methods, while the second assessment aims to assess the robustness of risk-active pathways and genes predicted by these methods. The mean reproducibility power and total number of identified informative pathways and genes were evaluated. Based on the first assessment, the mean reproducibility power of pathway activity inference methods generally decreased as the number of pathway selections increased. Entropy-based Directed Random Walk (e-DRW) distinctly outperformed other methods in exhibiting the greatest reproducibility power across all cancer datasets. On the other hand, the second assessment shows that no methods provide satisfactory results across datasets.

**Conclusion:**

However, PTB methods generally appear to perform better in producing greater reproducibility power and identifying potential cancer markers compared to non-TB methods.

**Supplementary Information:**

The online version contains supplementary material available at 10.1186/s12859-024-05632-w.

## Background

The emergence of high-throughput technologies facilitates the measurement of gene expression levels of tens of thousands of genes in the scope of a single experiment [1, 2]. Most of these experiments involve the comparisons of gene expression patterns across groups/classes, such as cases vs. controls or exposed vs. unexposed. Such comparison between phenotypes seeks to identify diagnostic markers of various disease states, outcomes, or responses to treatment [3]. Various differential expression analyses evolved to identify genes that may have roles in a given phenomenon or phenotype. These analyses typically yield a list of differentially expressed genes or proteins computed based on test statistics/p-values (e.g., T-test, Z-score, fold change, ANOVA, etc.) [4–7]. Although such lists of genes effectively differentiate between phenotypes, it fails to provide mechanistic insights into the underlying complex mechanisms involved in a given condition [8, 9]. The selection of differentially expressed genes (DEGs) is often subjective and these DEGs are only mapped to a small fraction of pathways [10]. This results in the exclusion of many highly expressed genes from pathway level analyses and does not elucidate pathway activities as a whole.

Another challenge in the analysis of genome-wide expression profiles is the robustness of individual gene biomarkers identified in microarray gene expression analysis. The prediction performance of identified gene markers in one dataset often decreased drastically when applied in an independent dataset of the same disease phenotype [11, 12]. This variation is typically due to the cellular heterogeneity within tissues, the inherent genetic heterogeneity across patients, and the measurement error in microarray platforms [13]. In addition, the large dimension small sample size problem and the redundant information produced from independent selection of gene markers further deteriorate the classification and prediction performance [14]. Hence, it is crucial to transform the gene-level results into a broader biological context to obtain a global view of expression changes and identify robust biomarkers at the level of functional categories. It is also much easier to investigate variations of samples at the pathway level rather than gene level to generate abstract quantification of pathways for characterising underlying biological mechanisms [15, 16].

One of the most common approaches used to address this goal is by grouping the long lists of individual genes into smaller sets of function-related genes or proteins [9]. Such an approach is known as Gene Set Analysis, or commonly referred to as Pathway Analysis (PA). In PA methods, knowledge bases (i.e. database collections of molecular knowledge) are utilised to aggregate genes into gene sets that share similar biological or functional properties. The resultant gene sets are analysed as a whole to identify which of these properties are relevant to the phenotype of interest [3]. PA methods overcome the limitations of interpreting overwhelmingly long lists of significant but isolated genes removed from biological context in differential expression analysis [17]. By leveraging the knowledge contained in various pathway databases (e.g. Kyoto Encyclopedia of Genes and Genomes (KEGG) [18], Reactome [19], NCI-PID [20], WikiPathways [21], etc.), it aims to detect pathways significantly enriched between two experimental conditions [22]. The activity of groups of biologically related genes rather than individual genes are analysed to investigate sample-wise variations at the pathway level.

Traditional PA methods treat pathways as unstructured gene sets and define pathway activity as the enrichment of the pathway genes among the top detections. These methods are commonly referred to as non-Topology Based (non-TB) methods, or Gene Set Analysis methods. Non-TB methods discard a substantial amount of knowledge regarding the positions and roles of the genes within the pathways, as well as the directions and types of the signals transmitted from one gene to another [8]. Another modern PA methods called Pathway Topology Based (PTB) methods have been developed in an attempt to include all this biological knowledge in analysis. It considers the underlying graphical structure or pathway topology when determining pathway activities. Such approaches model the whole biological system as networks, in which nodes represent related genes or proteins, and edges indicates interactions among them based on prior knowledge [8]. This enrichment analysis has been achieved by coupling pathway databases with statistical testing, mathematical analyses, and computational algorithms [23].

Although PA methods have been developed and used for well over a decade, there still exist a limited number of formal assessments and comparisons of tools and algorithms. There are several reviews [17, 23] and benchmark [10, 22, 24, 25] articles published offering guidance on the selection of PA methods. Most of these review [9, 26–28] articles covered an overview of the existing PA methods, ranging from Non-TB methods to PTB methods. These published works mainly focused on their theoretical definitions or underlying statistical concepts. There are some studies [24, 29, 30] that extensively compared the performance of PA methods based on benchmark data. However, these comparative studies are limited to Gene Set Analysis Methods (Non-TB). The comparison between Non-TB and PTB methods are outside of the scope of these analyses. Additionally, some former surveys [8, 22] performed a wide range of assessment encompassing accuracy, sensitivity, specificity, and the area under the receiver operating characteristic curve (AUC). These studies do not take into account the robustness evaluations of PA methods.

In this study, a systematic comparison of the performances of seven different pathway activity inference methods on six microarray gene expression datasets are presented based on two assessments. The main focus of this work is to provide a meaningful comparison of established pathway activity inference methods in terms of their ability to (i) generate high reproducibility power (robustness of pathway activity), and (ii) identify potential pathway markers and gene markers based on reproducibility of predictions (robustness of predicted risk-active pathways and genes). For comparability of the methods, four Gene Set Analysis methods and three PTB methods were implemented in the R statistical computing environment (see Table 1). These seven methods represent both Non-TB and PTB approaches. The selection criteria are based on their mathematical basis to represent clearly different approaches, as well as their availability and functionality for applications. Besides, six gene expression data and pathway data are prepared for the robustness evaluations. Each of these methods and input data as well as the workflow of the two assessments will be described thoroughly in the following sections.

**Table 1:** General information on the tested pathway activity inference methods

Method	Description	Category	Pathway representation	References
COMBINER	Multi-level optimisation framework for core module inference	Non-TB	GS	[31]
PAC	Pathway activity inference scheme in a condition-specific manner	Non-TB	GS	[16]
PLAGE	Pathway level analysis of gene expression based on singular value decomposition (SVD)	Non-TB	GS	[32]
GSVA	Gene set enrichment based on Kolmogorov Smirnov-like random statistic	Non-TB	GS	[33]
DRW	Random walk restart on KEGG network	PTB	PT	[13]
sDRW	Random walk restart on KEGG network	PTB	PT	[34]
e-DRW	Bi-random walk restart on KEGG and NCI-PID network separately	PTB	PT	[35]

non-TB: non-topology based method, PTB: pathway topology based method; GS: gene set, PT: pathway topology.

To perform a fair comparison of different pathway activity inference methods, it was necessary to employ the gene expression data that are processed and filtered in the same way. The relevant data pre-processing steps are described in detail in the second section of Materials and Methods. As the integration of topological pathway data represents a key component in the analysis of pathway activity inference tools, the number of pathway data inputs were retained as implemented in the original article to ensure the objectivity of evaluations and maximise the performances of different pathway activity inference methods when analysing the large number of cancer datasets. The pathway data used for evaluations are provided in the third section of Materials and Methods. Moreover, all classification evaluations for the seven tested methods are fixed the same for an effective comparison of prediction performances. The relevant classification evaluations are elaborated in the Comparative Approach section.

## Results

This section presents the results of two comparative assessments for the seven tested methods across six cancer datasets. The first assessment evaluates and compares the mean reproducibility power of different pathway activity inference methods. The second assessment investigates the number of identified informative pathway markers and gene markers for each method.

### Robustness of pathway activity

**Fig. 1:**
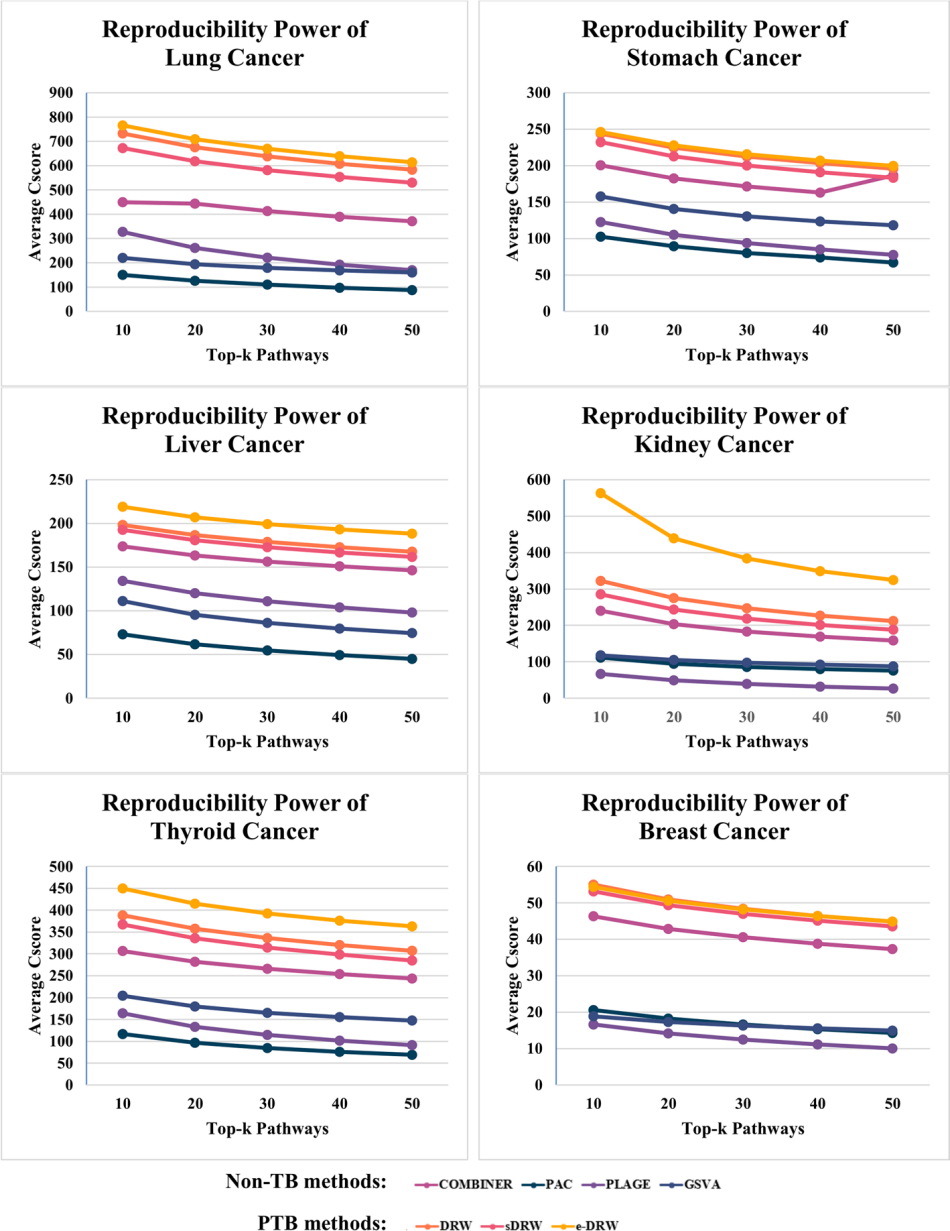
Comparison of mean reproducibility power for seven pathway activity inference methods

The selected pathway activity inference methods were applied to each of the six gene expression datasets, and the top-k active pathways (50, 40, 30, 20, 10) were selected for evaluations. The mean reproducibility power quantified using the Cscore method proposed by Yang et al. [31], of the top-k pathways for four Non-TB methods: COMBINER, PAC, PLAGE, GSVA, and three PTB methods: DRW, sDRW, and e-DRW were compared. Figure 1 shows the comparison of mean reproducibility power for seven pathway activity inference methods across all datasets.

Based on Fig. 1 above, the mean reproducibility power of pathway activity inference methods generally decreased as the number of pathway selections (Top-k pathways) increased—a trend observed for all six gene expression datasets. This observation reflects the dimensions of pathway selections can affect the reproducibility performance encountered with any methods. Notably, the PTB methods are significantly more robust than non-TB methods in generating greater reproducibility power. Specifically, the range of reproducibility power scores obtained by PTB methods (from 43 to 766) are much higher than non-TB methods (from 10 to 493) for all pathway selections across datasets. Among the non-TB methods, COMBINER consistently performs better than any other methods (PAC, PLAGE, and GSVA) for top-k pathway selections across six cancer datasets.

Compared individually with other pathway activity inference methods, e-DRW almost always produced the highest mean reproducibility power across all datasets except for breast cancer dataset, whereas PAC consistently produced the lowest mean reproducibility power for all pathway selections across majority of the datasets. This indicates that e-DRW exhibited the greatest power to discriminate between tumour and normal samples for all five datasets. On the other hand, DRW presented exceptionally high mean reproducibility power for top-40, 30, 20, 10 pathway selections in breast cancer dataset, although the reproducibility performances were slightly higher than e-DRW. Additional file 1 summarised the mean reproducibility power of each pathway activity inference methods across six cancer datasets, and Additional files 2, 3, 4, 5, 6, 7 corroborated the findings in detail for the seven tested methods.

**Fig. 2:**
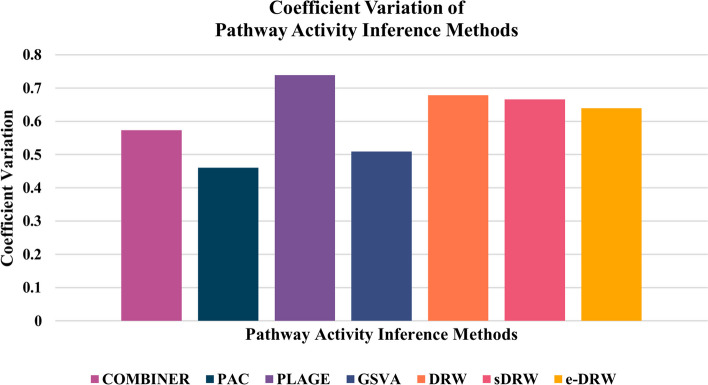
Coefficient variation of pathway activity inference methods

In addition, another comparison of reproducibility power based on coefficient of variation (CV) was conducted to evaluate the performance of pathway activity inference methods. CV was calculated as a statistical measure of the method’s robustness based on the ratio of the standard deviation to the mean [36]. The higher the CV, the greater the degree of dispersion around the mean. Based on the evaluations, three out of four non-TB methods (COMBINER, PAC, and GSVA) exhibited low CV (below 60%) compared to PTB methods which reported a higher degree of variation to its mean (above 60%). This indicates that the dispersion of reproducibility power scores for non-TB methods are much better than PTB methods across all datasets. Compared CV individually with other methods, PAC delivered the lowest CV whereas PLAGE generated the highest variability of reproducibility power scores. Figure 2 illustrates the CV of each pathway activity inference methods.

### Robustness of predicted risk-active pathways and genes

To assess the robustness of risk-active pathways and genes predicted by different pathway activity inference methods, classifier that produces the highest mean accuracy across majority of the cancer datasets for each method was chosen for further evaluations. For methods that generate comparable results across datasets, the classifier that predicts the highest number of pathways was selected for analysis. Additional file 8 summarised the mean classification accuracy of pathway activity inference methods across six cancer datasets using three different classifiers (NB, KNN, LR). Besides, Additional files 9, 10, 11, 12, 13, 14 details the mean accuracy and prediction results of selected classifier across 10 experiments for the seven tested methods. Table 2 outlines the number of predicted pathway markers for seven pathway activity inference methods across all datasets. The number of predicted pathway markers refers to the number of pathways determined by the seven computational methods after classification.

**Table 2:** Number of predicted pathway markers

Pathway activity inference methods	Gene expression dataset					
	Lung GSE10072	Stomach GSE13911	Liver GSE17856	Kidney GSE15641	Thyroid GSE33630	Breast GSE3494
COMBINER	10	12	13	18	14	22
PAC	13	18	32	15	19	22
PLAGE	41	16	20	8	37	8
GSVA	15	15	18	29	12	6
DRW	10	12	12	26	16	17
sDRW	11	8	13	20	10	20
e-DRW	8	13	7	12	9	12

**Fig. 3:**
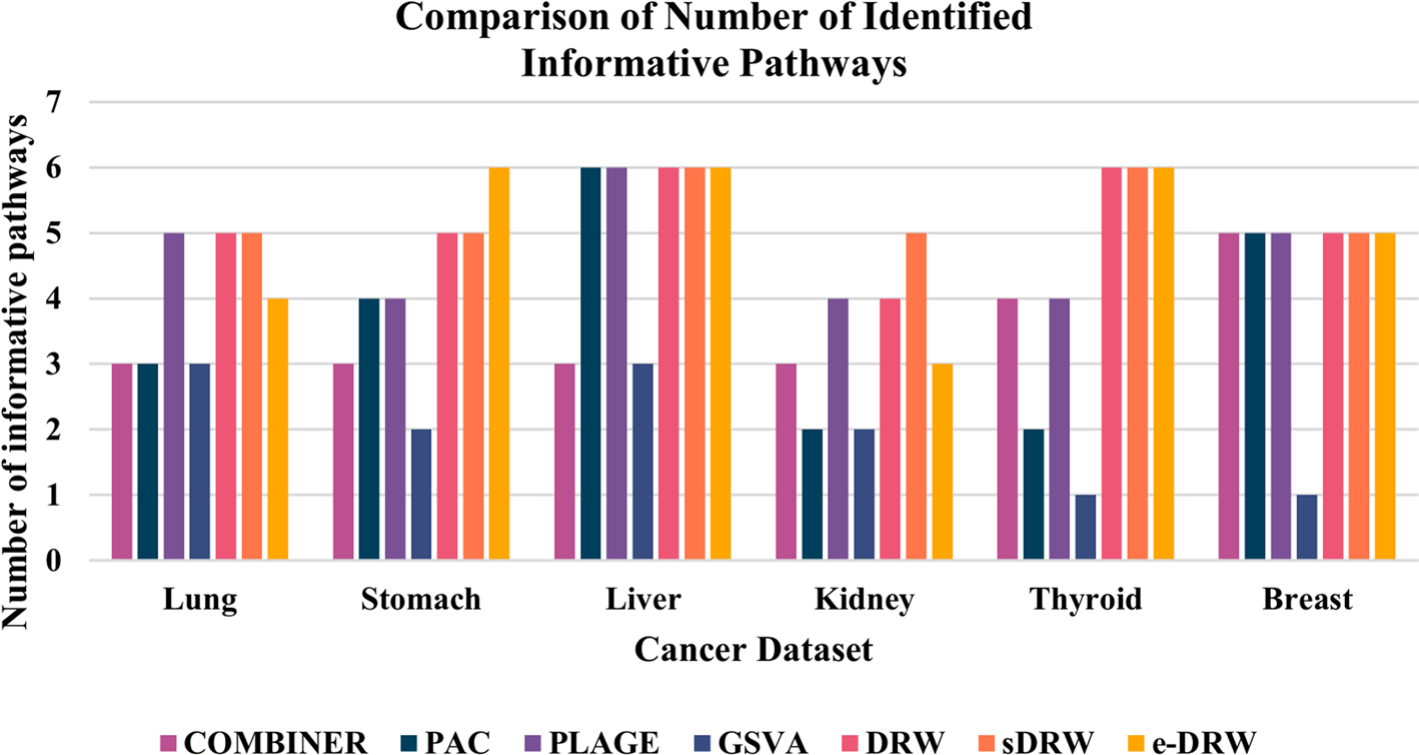
Comparison of number of identified informative pathways

Based on Table 2 above, PAC predicted the highest number of pathway markers across majority of the datasets compared to other pathway activity inference methods. However, highest prediction performance does not guarantee the robustness of predicted pathway markers. Thus, literature validation of pathways possesses a significant role to assess whether the candidate pathways is indeed associated not only with cancer, but also with other diseases or conditions. To ensure comparability of validation results between the computational methods, top-k pathways were selected from each method across all datasets based on the minimum number of predicted pathway markers (i.e. 6 pathway markers) as shown in Table 2. Figure 3 presents the number of identified informative pathways for seven pathway activity inference methods across all datasets. The number of identified informative pathways refers to the number of pathway (or gene) markers with PMIDs identified by PubMed text data mining.

**Fig. 4:**
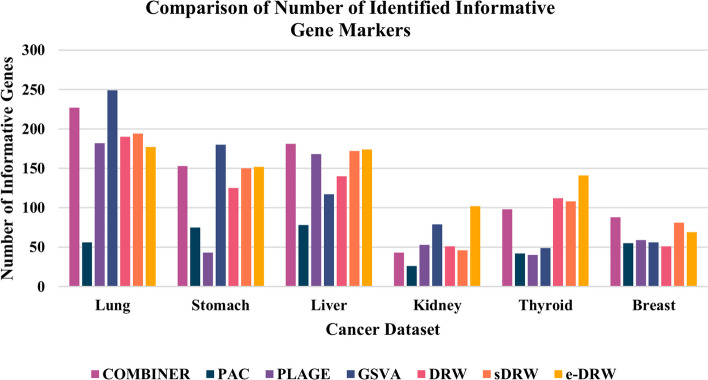
Comparison of number of identified informative genes

According to Fig. 3 above, PTB methods (DRW, sDRW, and e-DRW) evidently identified higher number of informative pathways compared to non-TB methods (COMBINER, PAC, PLAGE, and GSVA) across all datasets. Among the PTB methods, there is a subtle difference in performance for the identified informative pathway markers. Particularly, sDRW produced the highest number of identified informative pathways across five datasets except for stomach cancer dataset, whereas DRW and e-DRW each outperformed other pathway activity inference methods across four cancer datasets. In contrast, GSVA consistently identified the lowest number of informative pathway markers across all datasets. Among the non-TB methods, PLAGE encountered more identified pathway markers across three datasets, which are lung cancer, liver cancer, and breast cancer datasets. On the other hand, COMBINER, PAC, and GSVA generally produced lower number of identified pathway markers across majority of the datasets. GSVA turns out to deliver only one cancer pathway marker in thyroid cancer and breast cancer dataset which represents the lowest figure shown in the chart. Additional file 15 presents the top-6 frequently selected pathway markers with their PMIDs identified by PubMed text data mining. By selecting the top-6 frequently selected pathway markers, candidate genes were extracted from these risk-active pathways for further robustness evaluation based on PubMed text data mining. Figure 4 illustrates the number of identified informative gene markers for the seven pathway activity inference methods across all datasets.

Based on Fig. 4 above, there is a mixed findings reported from the evaluations. Notably, non-TB methods outperformed PTB methods by delivering highest number of identified informative gene markers across four datasets, which include lung cancer and stomach cancer datasets identified by GSVA, as well as liver cancer and breast cancer datasets detected by COMBINER. Whereas PTB method or specifically e-DRW performed better for kidney cancer and thyroid cancer datasets. In contrast, PAC consistently generated the lowest number of identified informative gene markers across four cancer datasets, ranging from lung cancer, liver cancer, kidney cancer, and breast cancer datasets. Whereas PLAGE identified the lowest informative gene markers for stomach and thyroid cancer datasets. Apart from that, DRW and sDRW delivered comparable results with subtle differences in performance. The identified informative gene markers are roughly proportional to each other across all datasets. Of the seven pathway activity inference methods, PLAGE identified the lowest figure in thyroid cancer dataset as shown in the bar chart. Additional files 16, 17, 18, 19, 20, 21 provides the genes in the frequently selected pathway markers (Top-6) with their PMIDs identified by PubMed text data mining across all datasets.

## Discussions

The goal of pathway level analysis is to transform a potentially large list of differentially expressed genes (hundreds or a few thousands) into a smaller list of meaningful biological phenomena. A wide range of PA methods have been proposed that focuses on the collective activity of genes within biologically relevant entities such as pathways. These approaches seek to investigate enriched pathways by measuring the pathway activities across given phenotypes. Although there have been few published works guiding users on the selection of these methods, they are collectively limited in the following ways: (i) several reviews only discussed the theoretical or methodological aspects of the methods; (ii) some comparative studies limited to the performance evaluations of non-TB methods, and (iii) majority of the surveys specifically focused on popular metrics (e.g. prioritisation, sensitivity, specificity, and accuracy) for performance evaluations. Thus, to address the aforementioned issues, this study provides a systematic assessment and comparison of seven widely used pathway activity inference methods (4 non-TB and 3 PTB methods) to evaluate the robustness of pathway activities and predicted cancer markers.

Based on the first assessment that evaluates the robustness of pathway activities in pathway activity inference methods, decreasing the number of pathway selections steadily increased the performance of reproducibility power. This observation is due to the fact that as the dimensions of pathway selections decreased, statistically significant pathway activities (high absolute t-scores) were selected from training-test pair datasets for evaluations. Pathway activities are considered reproducible if it provides similar discriminative power on both datasets. Besides, although each method attained different mean reproducibility power scores for different datasets, which presumably reflects the disparate biological processes represented in each dataset, it can be clearly observed that PTB methods consistently ranked higher than non-TB methods across all datasets. In particular, e-DRW was always ranked highest, followed by DRW and sDRW, whereas PAC and PLAGE fell to a low rank as seen in Fig. 1. This could be attributed to the construction of pathway topology and the robust gene-weighting method proposed by PTB methods. Comprehensive pathway topology helps clarify the roles that genes play in the pathway and weigh the genes more precisely. It also enables a more accurate prediction of disease status in PTB methods [13]. In addition, the application of gene-weighting method based on topological importance further maximises the ability of PTB methods to discriminate between tumour and normal samples compared to non-TB methods.

Moreover, based on the coefficient variation of mean reproducibility power evaluated on the seven tested methods across datasets, non-TB methods surprisingly performed better than PTB methods. This reflects the relative variability of reproducibility power scores produced by non-TB methods provides a more stable and precise performance across different cancer datasets. However, although non-TB methods generated robust performance across all datasets, the reproducibility power scores were steadily low compared to PTB methods. Hence, the reproducibility performance of PTB methods were still favourable as it exhibited greater robustness and discriminative power of pathway activity. On the other hand, based on the second assessment that evaluates the robustness of risk-active pathways and genes predicted by seven pathway activity inference methods, it was complicated to conclude the robustness performances as there was no outstanding methods that successfully delivered favourable outcomes in both the number of identified informative pathway markers and gene markers. However, PTB methods appear to outperform non-TB methods for the number of identified informative pathway markers across datasets. Specifically, sDRW performed well across five cancer datasets, while GSVA constantly detected lowest figures as shown in Fig. 3. The reliable performance of PTB methods could be attributed to the efficient pathway scoring incorporated in classification. The pathway activity inference schemes proposed by PTB methods effectively capture the biological interpretation of gene expression in functional categories and predicted reproducible pathway markers across datasets.

Furthermore, according to the robustness evaluations of predicted risk genes, there was a disparate result obtained from the assessment. In particular, non-TB methods successfully outperformed PTB methods across four datasets, which include lung cancer and stomach cancer datasets identified by GSVA, as well as liver cancer and breast cancer datasets detected by COMBINER. Whereas kidney cancer and thyroid cancer datasets were effectively identified by PTB method (e-DRW). The possible reason could be due to the pathway size utilised in the experiments. GSVA, COMBINER, and e-DRW each employed larger pathway sample size (see Table 4) compared to other methods. Not surprisingly, having more complete biological pathway information not only increases the method’s performance, but also enables a more accurate prediction of informative biomarkers for clinical utility towards prediction and treatment [13, 16, 37]. In contrast, PAC consistently produced the lowest number of identified informative gene markers across majority of the datasets. This is possibly due to the ignorance of structure information embedded in the pathway network. PAC disregards member genes that have consistent, but low-level expression changes under different phenotypes [38]. Thus, a flexible pathway topological information mining method is critical to produce reliable pathways and biomarkers for further diagnosis and prognosis applications.

To choose the best pathway activity inference methods, some guidance is provided to researchers based on the extensive assessments and comparisons. PTB methods provide better ability in generating greater reproducibility power and identify potential pathway markers. It was recommended for applications as the topology-based approach not only reflects the interactions between genes at the network level, but also considers the perturbation of high connectivity hub genes on pathways [39]. Besides, the scoring of pathway activity adopted by PTB methods effectively disregards genes with little topological importance to compute the activity of the pathways or subnetworks. Conversely, the results of non-TB methods are not very suitable in the context of pathway analysis. Although it demonstrates the capabilities in the identification of potential gene markers, the performance was highly dependent on the coverage of human pathway information. Non-TB methods treated all genes in the pathway equally and consider rather simple summary of expression values of the member genes for pathway activity inference [38].

## Conclusion

In this study, robustness evaluations of pathway activity inference methods are presented based on two assessments: (i) robustness of pathway activity based on reproducibility power, and (ii) robustness of predicted risk-active pathways and genes based on number of identified informative cancer markers. Reproducibility power metric quantifies the robustness of pathway activities, which aids in assessing the strength of different pathway analysis methods in discriminating between tumuor and normal samples or between different cancers. Besides, the number of identified informative pathway markers and gene markers assess the ability of pathway activity inference methods in identifying potential cancer markers that aid in future predictive and personalised medicine. Experimental results illustrated the feasibility of Pathway Topology-Based methods consistently produce larger reproducibility power and robust informative cancer markers across majority of the gene expression datasets. This could be attributed to the construction of pathway topology, gene-weighting based on topological importance, as well as the pathway scoring method employed by different topology-based approaches. While the current work assesses the robustness of pathway activity inference methods on gene expression data may be too narrow to accurately reflect the broad pool of pathway analysis methods proposed by other researchers. Therefore, this study presented possible variability of enrichment results that assesses the inherent capability of different pathway analysis methods.

## Materials and methods

This section presents the materials and methods used to evaluate the performance of pathway activity inference methods. The mathematical basis and concepts of each of the seven tested methods are described shortly. Detailed descriptions of these methods can be found in the original articles. Four of these Gene Set Analysis (Non-TB) methods include: COMBINER [31], PAC [16], PLAGE [32], and GSVA [33]. The other three PTB methods consist of: DRW [13], sDRW [34], and e-DRW [35]. Moreover, the pre-processing of gene expression data and pathway data is presented in detail. The statistical measures used for performance evaluations are also provided in this section.

### Non-TB pathway activity inference methods

#### COMBINER

COMBINER (COre Module Biomarker Identification with Network ExploRation) is proposed as a pathway-based biomarker identification framework to identify core modules that are consistently differentially expressed as a whole in the data cohorts of interest [31]. It adopts Core Module Inference (CMI) method by considering CORGs from both up- and downregulation genes with the most discriminative power to infer consistent pathway activities and identify driver genes within the core module. In COMBINER, given a pathway *P*_*j*_ consists of DEGs *{g*_*1*_*, g*_*2*_*,…, g*_*nj*_*}* ranked by descending order of their absolute t-scores with their normalised expression values, the pathway activity can be defined as:

1 $$a\left( {P_{j} } \right) = \frac{{\mathop \sum \nolimits_{i = 1}^{nj} Z\left( {gi} \right)*sign\left( {Tscore\left( {gi} \right)} \right)}}{\sqrt j }$$

where *a(Pj)* is the pathway activity of differential expression genes with p-value $\le$ 0.05 in a two-tailed t-test, *Z(gi)* is the normalised expression values of gene *gi*, *sign(Tscore(gi))* is the sign function of the the t-test statistics of gene *gi* from a two-tailed t-test with equal variances. *j* denotes the number of differential expression genes in the pathway where the markers are limited to maximum size of 20 genes. The above calculations of pathway activity were implemented in R.

#### PAC

PAC (Pathway Activity inference using Condition-responsive genes) is proposed as a gene expression-based diagnostic by incorporating pathway information in a condition-specific manner. It is motivated by the fact that only a subset of genes in a pathway are DEGs rather than the whole [10]. Thus, the markers are encoded as subset of “condition-responsive genes (CORGs)” in the pathway whose combined expression delivers optimal discriminative power for the disease phenotype [16]. To construct the CORGs set, t-test scores are computed to rank the member genes in ascending order if the average t-score among all member genes was negative, and in descending order otherwise. Within each pathway *P*_*j*_, the pathway activities *a(P*_*j*_*)* of CORGs are defined as:

2 $$a\left( {P_{j} } \right) = \frac{{\mathop \sum \nolimits_{i = 1}^{k} Z\left( {gij} \right)}}{\sqrt k }$$

where *Z(gij)* is the normalised z-transformed score which for each gene *gi* have mean µ_i_ = 0 and standard deviation $\sigma$_i_ = 1 over all samples *gj*. *k* refers to number of member genes in the CORGs set, which is used in the denominator to stabilise the variance of the mean. The PAC’s pathway activity matrix was calculated by utilising the *gsva ‘zscore’* function in GSVA library of R.

#### PLAGE

PLAGE (Pathway Level Analysis of Gene Expression) is a pathway-based method that works by transforming gene expression levels into pathway activity levels based on SVD strategy [32]. It begins by standardising gene expression profiles into z-scores over the samples and then calculates the SVD on the z-scores of the genes in the gene set. For each pathway *P*_*j*_, the pathway activity *a(P*_*j*_*)* at a single pathway-level value can be computed by:

3 $$a\left( {P_{j} } \right) = UDV$$

where *U* is a *m x n* matrix, *D* is a *n x n* diagonal matrix, and *V* is also a *n x n* matrix. The columns of *U* are known as the left singular vectors that used as an eigensample. The rows of *V* contain the elements of the right singular vectors that used as an eigengene. The coefficients of the first right-singular vector (first column of *V*) are taken as the gene set summaries (pathway activities) of expression over the samples. This is aimed to capture both pathway activity at the level of a single sample and the component that contributed most to the total variance. Practically, *gsva ‘plage’* function in GSVA library of R was utilised for implementation.

#### GSVA

GSVA (Gene Set Variation Analysis) is a non-parametric and unsupervised Gene Set Enrichment (GSE) method that estimates the variation of pathway activity over a sample population [33]. It works analogously by calculating sample-wise gene set enrichment scores as a function of genes inside and outside the gene set to a competitive gene set test. It then further estimates the variation of gene set enrichment over the samples independently of any class label by using Kolmogorov Smirnov (KS)-like random statistic. This method can be conceptualised as a transformation of coordinate systems for gene expression data, from genes to gene sets. The GSVA pathway enrichment scores are calculated by:

4 $$ES_{jk}^{diff} = \left| {ES_{jk}^{ + } } \right| - \left| {ES_{jk}^{ - } } \right| = max_{l = 1, \ldots ,p} \left( {0, v_{jk} \left( l \right)} \right) - min_{l = 1, \ldots ,p} \left( {0, v_{jk} \left( l \right)} \right)$$

where $ES_{jk}^{ + }$ and $ES_{jk}^{ - }$ are the largest positive and negative random walk deviations from zero, respectively, for sample *j* and gene set *k*. The above calculation procedures were implemented using gsva function by default in GSVA library of R.

### TB pathway activity inference methods

#### DRW

DRW (Directed Random Walk) is aimed to capture the topological information embedded in global directed pathway network and infer a robust pathway activity for cancer classification. It considers directed edges in the network and utilises the strategy of weighting genes based on t-test statistics score to enhance the reproducibility of pathway activities. DRW starts random walker on a source node *s* (or a set of source nodes simultaneously). The walker transitions from its current node to a randomly chosen neighbour (based on edge weights) at each time step, or returns to source node *s* with probability *r*. DRW with restart is defined as:

5 $$W_{t + 1} = \left( {1 - {\text{r}}} \right)M^{T} W_{t} + {\text{r}}W_{0}$$

where *W*_*t*_ is a vector which the *i*^−th^ node holds the probability of being at node *i* at time, *t*. *M* is the row-normalised adjacency matrix of the graph, *G*. Random walk is initiated by assigning the initial probability vector, *W*_*0*_ to each node whose initial probability was 0. *W*_*0*_ is an absolute t-test score, which will be further normalised into a unit vector [5]. The restart probability *r* was set as 0.7. *W*_*t*_ converges to a unique steady state in the presence of the ground node. This was obtained by performing the iteration until the normalisation fall between *W*_*t*_ and *W*_*t*+*1*_ < 10^–10^. For each pathway, those genes that are differentially expressed with p-values less than 0.05 are selected to construct the pathway activity [13]. The pathway activity score of pathway *P*_*j*_ is calculated as follows:

6 $$a\left( {Pj} \right) = \frac{{\mathop \sum \nolimits_{i = 1}^{nj} W\infty \left( {gi} \right)*sign\left( {Tscore\left( {gi} \right)} \right)*Z\left( {gi} \right)}}{{\sqrt {\mathop \sum \nolimits_{i = 1}^{nj} (W\infty \left( {gi} \right))^{2} } }}$$

where *a(Pj)* is the pathway activity (or expression value vector), *W*_*∞*_ is the output of genes (or weight vector), *Tscore(gi)* is the t-test statistics of gene *gi* from a two-tailed t-test with equal variances on expression values between two classes, *z(gi)* is normalised value vector of gene *gi* across all the dataset, and *sign()* is the sign function that returns (+ 1) for positive numbers and (-1) for negative numbers. The above same procedures were implemented in R as described in the original article.

#### sDRW

sDRW (significant Directed Random Walk) is aimed to improve the accuracy and sensitivity of cancerous gene predictions in conventional DRW. It improves DRW by tuning the parameter selection in formula (5) in order to identify the optimal restart probability for selected cancer datasets. An additional weight variable has also been added to enhance the connectivity between nodes for cancer classification. sDRW developed by Seah et. al. [34] starts random walker from a single node. At every time step, the walker transitions from its current node to a randomly selected neighbour (based on edge weights) or goes back to previous node with probability *r*. *r* can vary according to the datasets due to the attraction of nodes [34]. sDRW can be defined as:

7 $$W_{t + 1} = \left( {1 - {\text{r}}} \right){\text{M}}\left( {\frac{{N_{1} + N_{2} }}{2}} \right) + {\text{r}}W_{t}$$

where, *W*_*t*_ is a vector of *i* node which is transmitted from *i-1* node while *M* is an adjacency matrix developed from the original directed graph (with edges) to a more strongly connected directed graph. N1 and N2 represent the weight of two connected nodes implemented in the equation. sDRW calculates significant pathway activities from pathway expression profiles based on formula (6) for cancer classification. The above similar procedures were computed in R based on the original article, except for the restart probability parameter *r* which was set to 0.7 as the classification performance did not change much with the change in the value of restart probability *r* [13].

#### e-DRW

e-DRW (entropy-based Directed Random Walk) is aimed to enhance the accuracy of conventional DRW by introducing a more robust gene weighting strategy and incorporates entropy metric to perform random walk process. It enhances the coverage of human pathway information by constructing two input networks (i.e. KEGG and NCI-PID networks) for efficient pathway activity inference. The proposed gene weighting method utilises the combination of Point Biserial Correlation (PBC) coefficients and t-test values to run the algorithm. In e-DRW, a random walker begins from a single node and transits from its current node either to another randomly selected neighbour (forward) node based on the edge weights or returns to the previous node with probability *r*. *r* was set between 0.1–0.9 to discover the best restart probability correspond to each cancer datasets. e-DRW on KEGG and NCI-PID networks can be defined as:

8 $$H_{t + 1} = \left( {1 - {\text{r}}} \right)E^{T} H_{t} + {\text{r}}H_{0}$$

where *H*_*t*_ represents transition probability of *i*^*th*^ node which is transmitted from *i-*_*1*_ node. *H*_*0*_ is the initial entropy probability vector and *E*^*T*^ is an adjacency matrix developed from the original directed graph (with edges) and *H*_*t*+*1*_ denotes the final entropy probability vector. This was obtained by performing the iteration until the normalisation fall between *H*_*t*_ and *H*_*t*+*1*_ < 10^–10^. To infer the activity score for each pathway, e-DRW pathway activity inference method is computed as follows:

9 $$a\left( {Pj} \right) = \frac{{\mathop \sum \nolimits_{i = 1}^{nj} H\infty \left( {gi} \right)*PCTscore\left( {gi} \right)*Z\left( {gi} \right)}}{{\sqrt {\mathop \sum \nolimits_{i = 1}^{nj} \left( {H\infty \left( {\frac{1 - gi}{{sum\left( {1 - gi} \right)}}} \right)} \right)^{2} } }}$$

where *a(P*_*j*_*)* is the pathway activity of pathway *P*_*j*_, *H*_*∞*_ is the output of genes (or weight vector), *PCTscore(gi)* is the summation of PBC between gene *gi* and class label (normal and tumour samples), and t-test statistics of gene *gi* from a two-tailed t-test with equal variances on expression values between two classes. *z(gi)* is normalised value vector of gene *gi* across all the dataset, and $H\infty \left( {\frac{1 - gi}{{sum\left( {1 - gi} \right)}}} \right)$ is the entropy weight of gene *gi*. Practically, the eDRW library of R was applied for pathway activity calculation and the restart probability parameter *r* was set to 0.7 as opposed to original parameter value settings (0.1–0.9) for comparability with other PTB methods.

### Gene expression data

Six gene expression datasets were obtained from the National Centre for Biotechnology Information (NCBI) Gene Expression Omnibus (GEO) database, which are lung [40], stomach [41], liver [42], kidney [43], thyroid [44], and breast [45] cancer datasets. The collected raw gene expression datasets undergo data pre-processing based on the method proposed by Hui et. al. to remove missing values, noisy data, incomplete data, and inconsistent data for performance evaluations of pathway activity inference methods [46]. The data pre-processing method consists of three phases: (i) data cleaning and imputation, (ii) normalisation of gene expression data, and (iii) data filtering. In the first phase, data cleaning involves removing the unwanted and empty values of attributes in raw gene expression datasets. Then, mean imputation was implemented to fill in the rows with incomplete values of attributes. Before proceeding to the next phase, data rearrangement was run through to prepare an organised data used for pathway activity inference. In the second phase, data normalisation was carried out using Gene Pattern to tune the gene expression data into a proper format suitable for analysis [47]. Data filtering was further conducted in the last phase to remove redundant features and reduce the size of the gene expression datasets. Table 3 shows the details of the selected gene expression datasets after pre-processing.

**Table 3:** Gene expression datasets after pre-processing [46]

Cancer dataset	GEO ID	Platform ID	Number of cancerous sample	Number of normal sample	Number of genes	
					Raw	Cleaned
Lung	GSE10072	GPL96	58	49	22,283	12,986
Stomach	GSE13911	GPL570	38	31	54,675	12,419
Liver	GSE17856	GPL6480	43	44	25,075	13,802
Kidney	GSE15641	GPL96	69	23	22,283	11,593
Thyroid	GSE33630	GPL570	60	45	54,675	12,986
Breast	GSE3494	GPL96	60	176	22,283	12,986

### Pathway data

Real pathway data available from public resources were used in this work. For Non-TB methods, the gene sets were obtained from Molecular Signature Database (MSigDB) C2 collection [48]. The curated gene sets are divided into two subcollections: Chemical and genetic perturbations (CGP) and Canonical pathways (CP). To integrate gene sets into pathway activity inference methods, *msigdbr* [49] software R-package was applied to import the human pathway data and convert the gene sets into simple lists of genes. On the other hand, PTB methods require specific pathway topology inputs for enrichment analyses. Thus, KEGG and NCI-PID pathway data were collected from their respective pathway databases to construct directed pathway networks for analysis. *NetPathMiner* [50] software R-package was utilised to transform KEGG pathways into KEGG network. Subsequently, *PaxtoolsR* [51] software R-package was applied to convert PID pathways into PID network. The constructed directed pathway networks consist of nodes and edges where each node in the graph represented a gene, while each directed edge represented how the genes interacted and controlled each other. The directions of the edges were determined by the type of interaction between the two genes found in both KEGG and PID pathway databases. Table 4 presents the pathway data used by each pathway activity inference methods for evaluation analysis.

**Table 4:** Pathway data used for each method in benchmark analysis

Method	Number of pathway input	Pathway database	Directed pathway network
COMBINER	624	MsigDB	–
PAC	472	MsigDB	–
PLAGE	400	MsigDB	–
GSVA	3225	MsigDB	–
DRW	300	KEGG	6618 nodes and 111,730 edges
sDRW	300	KEGG	6618 nodes and 111,730 edges
e-DRW	536	328 KEGG, 208 NCI-PID	KEGG: 6667 nodes and 116,773 edges, PID: 2817 nodes and 39,289 edges

### Statistical measures

#### Reproducibility power

Reproducibility power metric was proposed by Yang et. al. [31] to measure the consistency or the degree of correlations of pathway activities between different datasets for assessing the robustness of pathway activity inference methods. Based on the principle proposed, the higher the reproducibility power, the stronger the robustness and discriminative power of pathway activity [16, 22]. The reproducibility power is shown as below:

10 $$Cscore\left( N \right) = \frac{1}{N}\mathop \sum \limits_{i = 1}^{N} tscore\left( {P\begin{array}{*{20}c} i \\ T \\ \end{array} } \right)*tscore\left( {P\begin{array}{*{20}c} i \\ V \\ \end{array} } \right)$$

where *tscore(P)* is the t-scores of *P* from a two-tailed T-test with equal variances on pathway activities between two classes, $$P\begin{array}{*{20}c} i \\ T \\ \end{array}$$ is the *i*-th pathway activity (ranked by absolute t-scores in descending order) in the training dataset, $$P\begin{array}{*{20}c} i \\ V \\ \end{array}$$ is its corresponding pathway activity in the test dataset, and *N* is the number of selected pathways.

### Number of identified informative pathways and genes

**Fig. 5:**
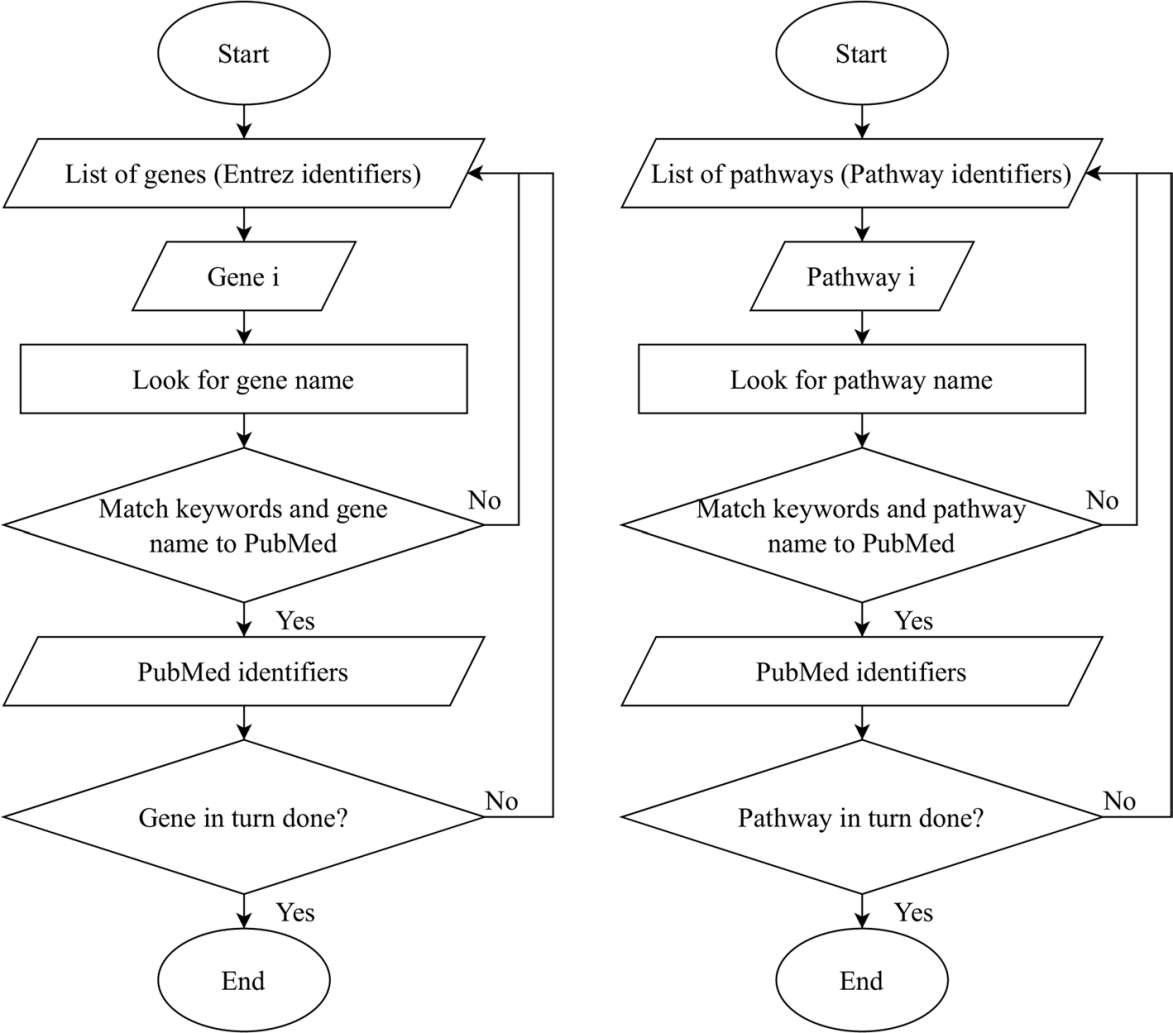
PubMed text data mining automation based on pathways and genes [52]

Number of identified informative pathway markers and gene markers are statistical measures proposed by Nies et. al. [52] to assess the ability of pathway activity inference methods in identifying potential cancer markers. PubMed text data mining automation was developed as the text mining technique to extract potential prognostic markers from scientific articles in PubMed database. This technique explores the relationships between pathways, genes, and cancers (pathway-disease and gene-disease relationships) based on Natural Language Processing (NLP). The basic concept of PubMed text data mining automation takes a list of genes (or pathways) as input and matches the keywords defined to PubMed database. The main keyword terms to be extracted include "pathway name", "gene name", "prognostic", and "cancer types" [52]. This concept was employed to illustrate the pathways and genes that exhibit biological traits related to cancers [52]. The keyword cancer markers specific to each cancer type include “Lung Cancer”, “Gastric Cancer”, “Hepatocellular Cancer”, “Renal Cell Cancer”, “Thyroid Cancer”, and “Breast Cancer”. During the mining process, disease-related text data in the PubMed database was optimised while the text data that are not related to biomarkers (or pathways) and diseases were ignored. Thus, PubMed identifiers (PMIDs) were acquired as a proof to determine the connection between pathways, genes, and diseases [53, 54]. The total number of pathway markers and gene markers with identified PMIDs were calculated to reflect the robustness of risk-active pathways and genes predicted by each pathway activity inference methods. Figure 5 illustrates the process flow of PubMed text data mining [52].

In the keywords matching process, this technique utilises easyPubMed R package to retrieve data from PubMed database. It automatically queries PubMed records from the Entrez History Server for an easy and smooth programmatic access [55]. If the genes or pathways do not match the keywords with the PubMed database, the mining process will continue to look for the following genes or pathways [52]. The entire process is repeated until each and every gene and pathway is verified and validated.

### Comparative approach

This section introduces two comparative assessments for performance evaluations of pathway activity inference methods. The first assessment is aimed to investigate the robustness of different pathway activity inference methods based on reproducibility power score. The second assessment focuses on evaluating the ability of the seven tested methods in identifying potential pathway markers and gene markers based on number of identified informative pathways and genes.

### Assessment 1: Robustness of pathway activity

**Fig. 6:**
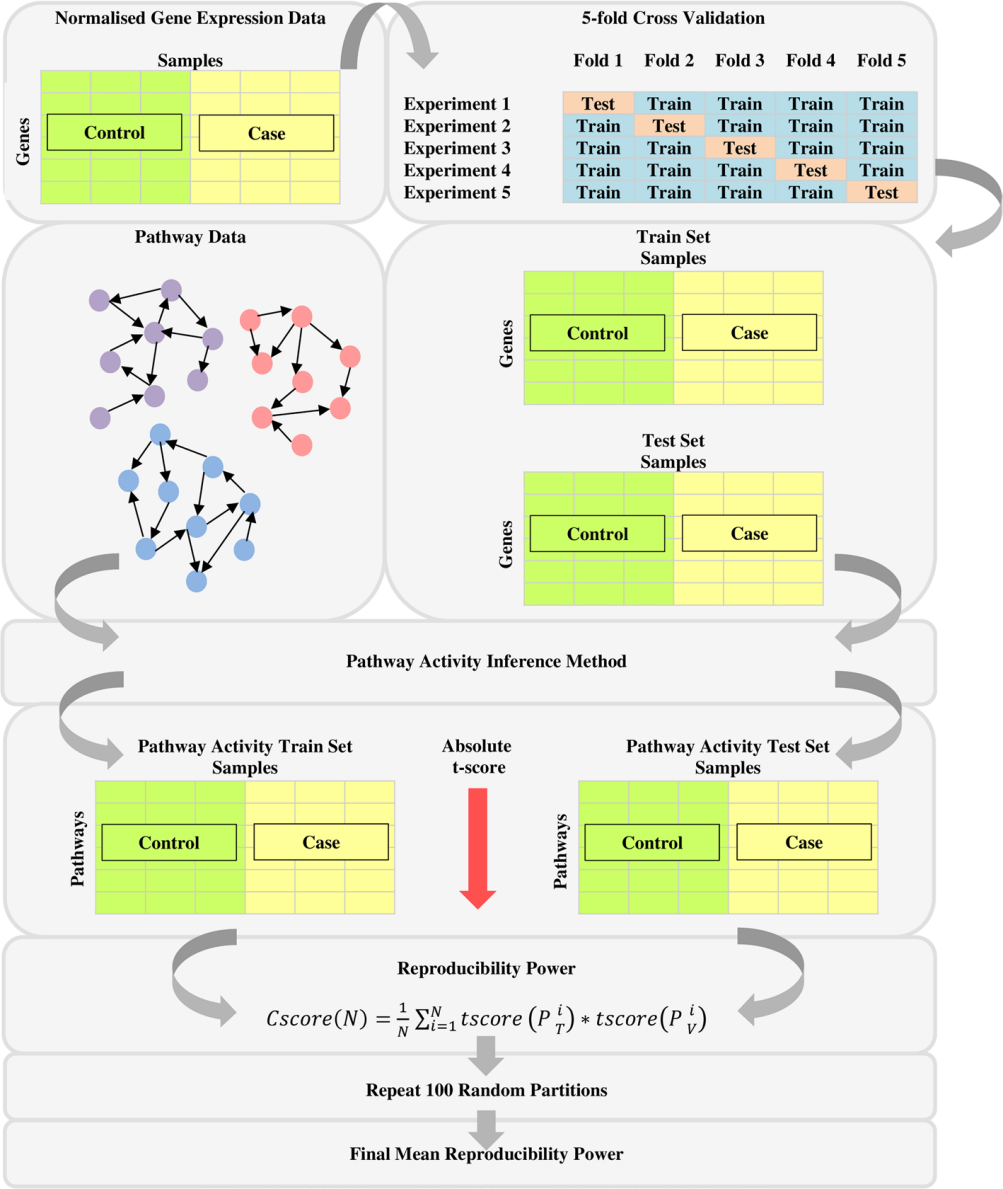
Workflow of evaluating pathway activity inference methods based on the robustness of pathway activity

To evaluate different pathway activity inference methods, reproducibility power metric was utilised to evaluate the robustness of pathway activities generated from each method. This metric measures the discriminative power and robustness between the pathway activities in training set and the pathway activities in test set [9]. To calculate the reproducibility power of pathway activity, the samples in normalised gene expression data begin by randomly divided into five subsets of equal size. Four of these subsets were used as the train set, whereas the remaining subset was used as the test set. Then, the train set, test set, and pathway data (either in the form of GS or PT) are supplied for the implementation of pathway activity inference methods. The enrichment analysis produces train set and test set pathway expression profiles for each experiment. After that, the reproducibility power of pathway activities were computed based on formula (10). Each subset was used in turn as the test set to evaluate the reproducibility. For unbiased evaluation, these experiments were repeated for 100 random partitions for the entire dataset. The mean reproducibility power (Cscore) over 500 experiments were reported as the overall performance [13]. Figure 6 shows the workflow of evaluating pathway activity inference methods based on the robustness of pathway activity.

### Assessment 2: robustness of predicted risk-active pathways and genes

To evaluate the robustness of risk-active pathways and genes predicted by each method, normalised gene expression data were first split into three subsets whereby 60% of the datasets were used as the training set, 20% used as the validation sets, and another 20% used as the test sets. The three subsets and the prepared pathway data were then utilised for the implementation of pathway activity inference methods. The enrichment analysis produces pathway expression profiles of training set, validation set, and test set ranked by absolute t-test statistics in descending order. Then, within-dataset experiments proposed by Tay et. al. [35] were implemented for the seven tested methods across six cancer datasets. The R caret software package was applied to obtain the classification accuracy. Three classifiers were selected to evaluate the classification performance, which include Naïve Bayes (NB), K-Nearest Neighbours (KNN), and Logistic Regression (LR). The top 50 pathways in the training set were used as candidate features to build the model. Subsequently, pathways were added sequentially to train the classification model. The performances of the classifiers were measured based on accuracy calculated from confusion matrix. The added pathway marker was maintained in the feature set if the AUC increased, but was removed if otherwise [13]. This process was repeated for the top 50 pathway markers to optimise the classifier and to yield the best feature set. The performance of the optimised classifier was evaluated on the test set using pathway markers from the best feature set. This process was repeated 10 times to ensure unbiased evaluation and to estimate the variation of the accuracy. As the final step, the mean accuracy across 10 classifiers was estimated to represent the overall performance of the classification method.

**Fig. 7:**
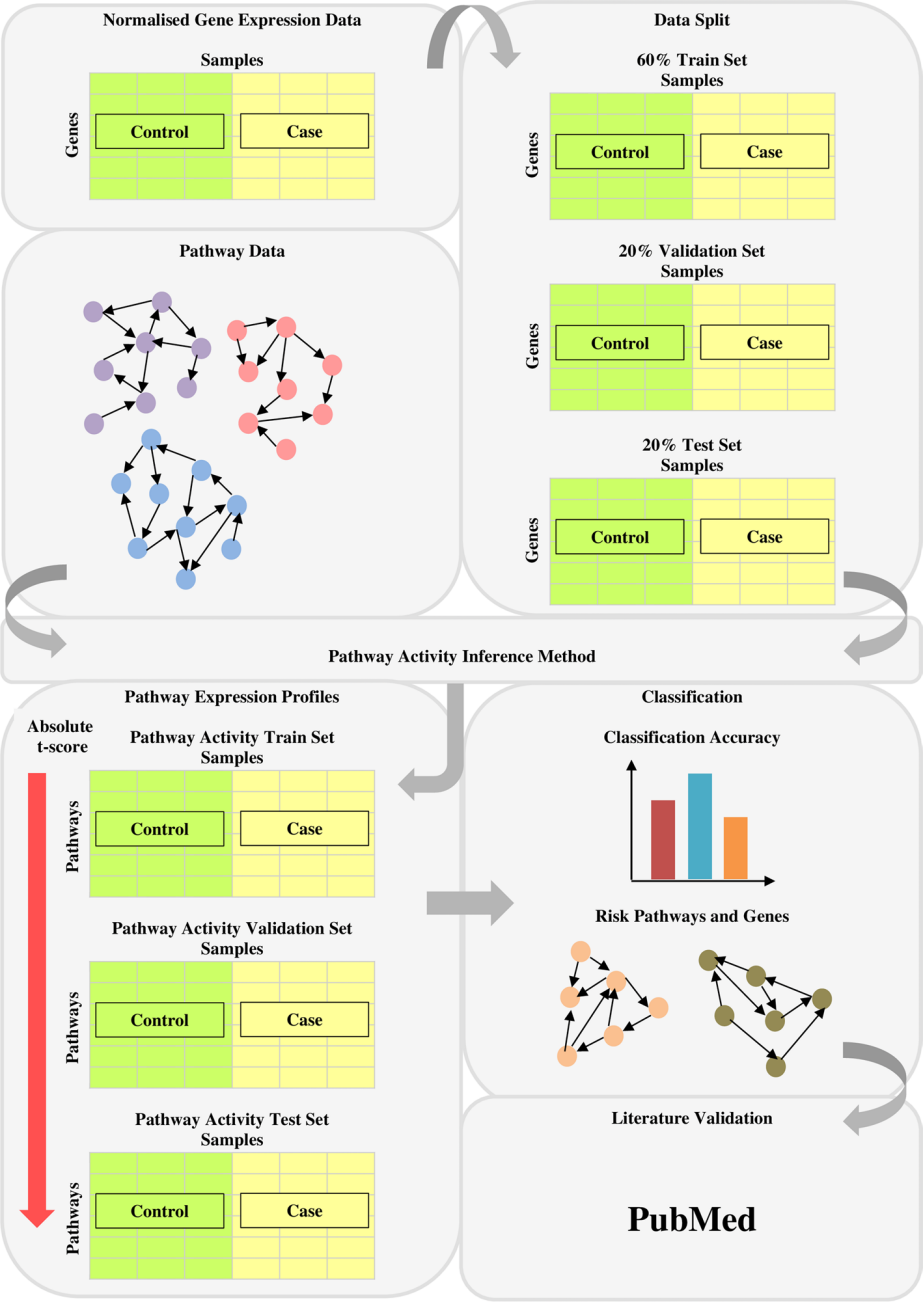
Workflow of evaluating pathway activity inference methods based on the robustness of predicted risk-active pathways and genes

After completing the classification evaluations for the seven tested methods, the classifier that produces the highest mean accuracy across majority of the cancer datasets for each method was chosen for further evaluations of predicted pathways and genes. Top-k frequently selected pathway markers across 10 experiments were chosen for literature validation based on PubMed text data mining. The process flow of evaluating the risk-active pathways and genes predicted by pathway activity inference methods is shown in Fig. 5. The total number of identified informative pathway markers and gene markers were calculated to statistically measure the robustness of prediction results as well as to assess their ability in identifying potential cancer markers. Figure 7 presents the workflow of evaluating pathway activity inference methods based on the robustness of predicted risk-active pathways and genes.

### Supplementary Information

**Additional file 1.** Mean reproducibility power of seven pathway activity inference methods across six cancer datasets.

**Additional file 2.** Mean reproducibility power of seven pathway activity inference methods for lung cancer dataset.

**Additional file 3.** Mean reproducibility power of seven pathway activity inference methods for stomach cancer dataset.

**Additional file 4.** Mean reproducibility power of seven pathway activity inference methods for liver cancer dataset.

**Additional file 5.** Mean reproducibility power of seven pathway activity inference methods for kidney cancer dataset.

**Additional file 6.** Mean reproducibility power of seven pathway activity inference methods for thyroid cancer dataset.

**Additional file 7.** Mean reproducibility power of seven pathway activity inference methods for breast cancer dataset.

**Additional file 8.** Mean accuracy of seven pathway activity inference methods across six cancer datasets using three classifiers.

**Additional file 9.** Classification accuracy and predicted risk-active pathways and genes of seven pathway activity inference methods for lung cancer dataset.

**Additional file 10.** Classification accuracy and predicted risk-active pathways and genes of seven pathway activity inference methods for stomach cancer dataset.

**Additional file 11.** Classification accuracy and predicted risk-active pathways and genes of seven pathway activity inference methods for liver cancer dataset.

**Additional file 12.** Classification accuracy and predicted risk-active pathways and genes of seven pathway activity inference methods for kidney cancer dataset.

**Additional file 13.** Classification accuracy and predicted risk-active pathways and genes of seven pathway activity inference methods for thyroid cancer dataset.

**Additional file 14.** Classification accuracy and predicted risk-active pathways and genes of seven pathway activity inference methods for breast cancer dataset.

**Additional file 15.** Literature validation of top-6 frequently selected pathway markers predicted by seven pathway activity inference methods across six cancer datasets.

**Additional file 16.** Literature validation of top-6 frequently selected pathway markers predicted by seven pathway activity inference methods for lung cancer dataset.

**Additional file 17.** Literature validation of top-6 frequently selected pathway markers predicted by seven pathway activity inference methods for stomach cancer dataset.

**Additional file 18.** Literature validation of top-6 frequently selected pathway markers predicted by seven pathway activity inference methods for liver cancer dataset.

**Additional file 19.** Literature validation of top-6 frequently selected pathway markers predicted by seven pathway activity inference methods for kidney cancer dataset.

**Additional file 20.** Literature validation of top-6 frequently selected pathway markers predicted by seven pathway activity inference methods for thyroid cancer dataset.

**Additional file 21.** Literature validation of top-6 frequently selected pathway markers predicted by seven pathway activity inference methods for breast cancer dataset.

## Data Availability

The data analysed in this paper are available in the Gene Expression Omnibus (GEO) repository at NCBI (https://www.ncbi.nlm.nih.gov/geo/query/acc.cgi?acc=GSE10072; https://www.ncbi.nlm.nih.gov/geo/query/acc.cgi?acc=gse13911; https://www.ncbi.nlm.nih.gov/geo/query/acc.cgi?acc=GSE17856; https://www.ncbi.nlm.nih.gov/geo/query/acc.cgi?acc=GSE15641; https://www.ncbi.nlm.nih.gov/geo/query/acc.cgi?acc=gse33630; https://www.ncbi.nlm.nih.gov/geo/query/acc.cgi?acc=gse3494).

## Abbreviations

PA: Pathway analysis
KEGG: Kyoto encyclopedia of genes and genomes
NCI-PID: National cancer institute nature pathway interaction database
DRW: Directed random walk
sDRW: Significant directed random walk
e-DRW: Entropy-based directed random walk
PAC: Pathway activity inference using condition-responsive genes
COMBINER: Core module biomarker identification with network exploration
NCBI: National centre for biotechnology information
GEO: Gene expression omnibus
CORGs: Condition-responsive genes
CMI: Core module inference
PMIDs: PubMed identifiers
Cscore: Reproducibility power
CV: Coefficient of variation
